# Immature Surfactant Protein Type B and Surfactant Protein Type D Correlate with Coronary Heart Disease in Patients with Type 2 Diabetes

**DOI:** 10.3390/life14070886

**Published:** 2024-07-17

**Authors:** Cristina Banfi, Francesco Piarulli, Eugenio Ragazzi, Stefania Ghilardi, Arianna Greco, Annunziata Lapolla, Giovanni Sartore

**Affiliations:** 1 Unit of Functional Proteomics, Metabolomics and Network Analysis, Centro Cardiologico Monzino IRCCS, 20138 Milan, Italy; stefania.ghilardi@cardiologicomonzino.it (S.G.); arianna.greco@cardiologicomonzino.it (A.G.); 2 Department of Medicine-DIMED, University of Padova, 35122 Padova, Italy; francesco.piarulli@unipd.it (F.P.); annunziata.lapolla@unipd.it (A.L.); g.sartore@unipd.it (G.S.); 3 Studium Patavinum, University of Padova, 35122 Padova, Italy; eugenio.ragazzi@unipd.it

**Keywords:** surfactant protein type D, precursor surfactant protein type B, type 2 diabetes, cardiovascular disease

## Abstract

Background: Different specific surfactant proteins (SPs) have been associated with various pathological conditions, not only of the respiratory system, but also more recently with cardiovascular diseases, such as heart failure. The aim of the present study was to evaluate the role of SP-A, SP-D, and the precursor protein of SP-B (proSP-B) in the pathogenesis of cardiovascular damage in patients affected by type 2 diabetes (T2D). Methods: The study considered 31 patients with T2D (DN group), 34 patients with both T2D and coronary heart disease (CHD) (DC group), and 30 patients without diabetes but with a diagnosis of CHD (NC group). SP-A, SP-D, and proSP-B concentrations were determined in plasma samples, and were statistically compared using parametric and multivariate methods. Results: Higher plasma concentrations of SP-D and proSP-B were found in patients affected by both T2D and CHD (DC group), and in patients with CHD without diabetes (NC group), in comparison to T2D patients (DN group). A significant correlation, both with linear regression (*r* = 0.3565, *p* = 0.001) and Principal Component Analysis (PCA), was found between the plasma levels of SP-D and proSP-B in the overall cohort of patients. No differences in SP-A were observed among the three groups of subjects. Conclusion: The present study extends the knowledge on the role of plasma SPs’ levels as possible indicators of the risk of CHD being linked to T2D disease progression.

## 1. Introduction

Different specific surfactant proteins (SPs) have been described and subdivided into hydrophilic (SP-A and SP-D) and hydrophobic (SP-B and SP-C) types [1]. The surfactant hydrophilic proteins have been included among the C-type lectins group III, named as collectins. These proteins are thought to play a crucial role in the innate immune system, as they can detect various pathogens such as viruses, bacteria, fungi [2], and allergens [3]. SP-A has also been described in the structural organization of the surfactant, while SP-D contributes to surfactant homeostasis [1]. Additionally, SP-A is expressed in several extra-pulmonary tissues [4], including the blood vessel wall, where it acts as a regulator for smooth muscle cell phenotypic modulation [5]. Serum levels of SP-A have been suggested as a marker of pulmonary disease in smokers [6], and circulating SP-D levels have been proposed as a predictor of cardiovascular and pulmonary diseases [7,8]. A recent study has revealed that patients who exhibit a high level of circulatory SP-D, specifically those with levels ≥ 100 ng/mL, are more prone to severe peripheral artery disease [9], and are more likely to suffer from diabetes and tibial or peroneal artery stenosis/occlusion compared to their counterparts with lower SP-D levels. In addition, the study posits that there exists a correlation between SP-D levels and adverse outcomes, including cardiovascular death, heart failure, and leg amputation [10].

There is limited information available regarding the functional roles of surfactant protein SP-C within the hydrophobic group. Its main association is with the regulatory function and stability of the surfactant. Conversely, the properties of surfactant protein B (SP-B) have been investigated to a greater degree. It is a small protein (8 kDa of weight) composed of 79 amino acids (in particular valine, alanine, phenylalanine, leucine, isoleucine, and tryptophan), which assembles as a homodimer and has a pivotal role in stabilizing the alveolar surfactant [11,12], permitting the lung to inflate [11]. A hereditary deficiency of SP-B causes severe respiratory diseases [12], including acute respiratory distress, which is often fatal or followed by severe chronic lung disease.

SP-B is a protein primarily produced by the lung type II epithelial cells. Its biosynthesis is a complex process that involves both post-translational and proteolytic events. Initially, the protein is synthesized as a 40 kDa prepro-SP-B, which undergoes glycosylation and signal peptide cleavage to produce the 42-kDa pro-SP-B within the endoplasmic reticulum. The N-terminal propeptide of the protein is then cleaved in the medial Golgi, trans-Golgi, and possibly in a post-Golgi compartment, such as the multivesicular body. This ultimately results in the production of a mature form of SP-B in the lamellar bodies, which create a network of organized membranes, called tubular myelin, between the hypophase, a thin aqueous layer on the alveolar epithelial cell surface, and the air [13]. Precursor proteforms of SP-B (proSP-B) have been proposed as robust and adequate biomarkers for pulmonary diseases that impact the alveolo-capillary barrier [14,15,16,17,18]. In the intricate clinical context of heart failure (HF), pro-SPB plays a significant role; its increased levels have been linked to alveolar-capillary membrane dysfunction, and it has a potential prognostic value [19,20,21]. Additionally, it serves as an early indicator of the effectiveness of drug therapy [22,23].

People affected by diabetes have a two- to four-fold higher risk of developing coronary artery disease (CHD) than people without diabetes, according to Kannel and McGee, in the Framingham study [24]. In particular, prolonged duration of diabetes (>10–12 years) in men with diabetes increases CHD mortality at a rate similar to that of men without diabetes but with a history of prior myocardial infarction [25]. Cardiovascular disease is a common comorbidity in T2D, and CHD, together with stroke, are considered as major contributors [26]. Due to the possible implication of SPs in pathologies involving the cardiovascular system, it was thought to be of interest to evaluate the link of different specific SPs to coronary heart disease (CHD), which frequently afflicts patients with type 2 diabetes (T2D). The study used a previous enrollment procedure [27], considering a group of patients with T2D (DN group), a group of patients with both T2D and CHD (DC group), and another group of patients without diabetes but with a diagnosis of CHD (NC group). SP-A, SP-D, and proSP-B were measured in the plasma of the subjects, and were compared in order to verify any association between the proteins and the condition of coronary artery injury in the chronic evolution of T2D.

## 2. Materials and Methods

### 2.1. Patient Recruitment

The present cross-sectional observational study was conducted on the following three groups of patients: 34 patients who had both type 2 diabetes and coronary heart disease (group DC), 31 patients with T2D but not CHD (group DN), and 30 patients with CHD but no diabetes diagnosis (group NC). The protocol for patient selection derives from a previously published investigation [27]. All patients affected by diabetes (DC and NC groups) followed an isocaloric Mediterranean-style diet and received individualized hypoglycemic therapy, which consisted mainly of metformin (64% of DC patients, and 77% in DN patients, *p* = 0.23). CHD was defined according to standard criteria and anamnestic data from electronic medical and hospital records. Ninety percent of the CHD patients (NC group) were taking antihypertensive drugs. DC and NC patients were assuming low-dose aspirin (70% and 57%, respectively, *p* = 0.28) and statins (79% and 60%, respectively, *p* = 0.10). The patients in the three groups had similar smoking habits (47% to 55% were smokers/former smokers, *p* = 0.80). The study was conducted in the pre-COVID-19 period. All patients were in good overall health, without functional activity impairment, and with no signs of heart failure (dyspnea on exertion and/or rest, declivous oedema, NYHA class 0/I) or acute respiratory pathologies, and not suffering from neoplastic diseases. Patients with chronic obstructive pulmonary disease, asthma, history and/or documentation of pulmonary embolism and/or pulmonary hypertension, as well as patients with severe cerebrovascular, renal, hepatic, and hematologic diseases, were excluded from the study.

Clinical and metabolic parameters pertaining to the 3 groups of subjects are presented in Table S1. Full information regarding the patients’ cohort has been previously published [27]. The study was conducted in accordance with the Declaration of Helsinki, and was approved by the Ethics Committee for Clinical Trials in the Province of Padova, reference study No. 97610. Informed written consent was given by all participants.

### 2.2. Sample Preparation

Blood samples were collected in citrate tubes (0.129 mmol/L). The choice of citrate–plasma specimens derives from the recommendation of the HUPO Plasma Proteome Project (PPP) for the development and validation of circulating protein biomarkers in health and disease [28]. The plasma was immediately separated by centrifugation at 1500× *g* for 15 min at 4 °C, and then partitioned into aliquots, which were frozen at −80 °C until analysis.

### 2.3. Analysis of Surfactant Proteins

The immature form of SP-B (proSP-B) analysis was performed as previously described [19]. Briefly, in order to precisely resolve low-molecular weight proteins, equal amounts of plasma proteins (50 µg) were separated by one-dimensional Sodium Dodecyl Sulfate–Polyacrylamide Gel Electrophoresis (SDS-PAGE) on 15% polyacrylamide gels using a Tris-Tricine buffer system with no reducing conditions [29]. To ensure equivalent protein loading, the membranes were stained with MemCode^TM^ reversible protein stain from Pierce Biotechnology in Cramlington, UK. For each subject, the datum was reported as the ratio of band volume after local background subtraction to the volume of total proteins loaded and stained with MemCode. The values were normalized vs. the band volume of pooled plasma that was loaded as a control on each gel and expressed as arbitrary units (AU). An absolute concentration measurement was not possible, since the protein under investigation is not available in its pure form as a calibrant. The inter-assay coefficient of variation was 12.1 ± 2.9%. Plasma levels of SP-A and SP-D were determined using commercially available ELISA kits (BioVendor, Heidelberg, Germany). The intra-assay and inter-assay coefficients of variation for SP-A and SP-D were <5% and <10%, and <5.2% and <3%, respectively.

### 2.4. Statistical Evaluation of Data

Data were evaluated using JMP^®^ Version Pro 17 software for Windows (SAS Institute Inc, Cary, NC, USA) and JASP (JASP Team, 2023, Version 0.17.2). Values are expressed as means ± standard deviation (SD). To assess the statistical differences among groups, a preliminary one-way ANOVA followed by post hoc analysis with Tukey’s HSD test was used. Differences were considered statistically significant when *p* < 0.05. Correlation between variables was evaluated with linear regression, and the Pearson’s product moment correlation coefficient of *r* was calculated as a measure of linear association.

The non-parametric multivariate method of Principal Component Analysis (PCA) was also used [30] to obtain a summary based on fewer variables (scores) that represent the weighted average of the original variables, whose profiles are the loadings. Results were presented as a biplot [31], where the loading plot shows the correlations between variables, considering that when the angle between eigenvectors is near zero, it indicates a positive correlation, while angles of 90° indicate no correlation, and angles near 180° indicate negative correlations.

## 3. Results

Clinical and metabolic parameters of the three groups of subjects are presented in Table S1. Full characterization of the patients has been previously published [27].

**Figure 1:**
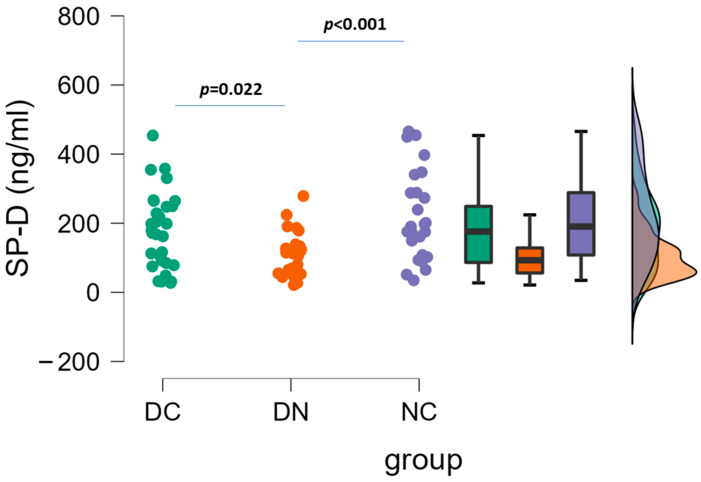
Plasma concentrations of SP-D in the three groups of patients. Raincloud data plot, as well as box plots, and theoretical distributions are reported. Statistical differences were assessed by one-way ANOVA, followed by post hoc Tukey’s test.

The plasma concentrations of SP-D in the three patient groups are depicted in Figure 1. By using one-way ANOVA followed by Tukey’s post hoc test, a significant difference was found between DC and DN groups (178.3 ± 110.8 vs. 103.9 ± 63.0 ng/mL, *p* = 0.022), and between DN and NC groups (103.9 ± 63.0 vs. 220.6 ± 129.9 ng/mL, *p* = 0.001). No significant difference occurred between DC and NC groups.

**Figure 2:**
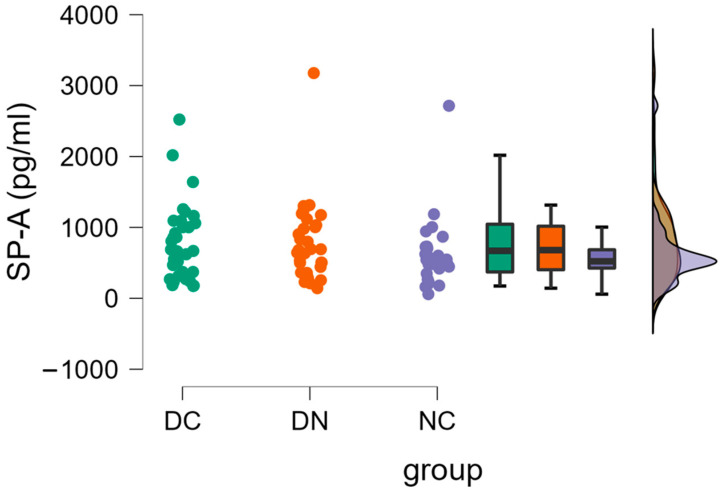
Plasma concentrations of SP-A in the three groups of patients. Raincloud data plot, as well as box plots, and theoretical distributions are reported. Statistical differences were assessed by oneway ANOVA.

Figure 2 indicates that there were no significant differences (one-way ANOVA) observed in the plasma concentrations of SP-A among the groups (DC: 782.5 ± 531.7 pg/mL; DN: 775.7 ± 569.1 pg/mL; NC: 604.6 ± 472.7 pg/mL).

**Figure 3:**
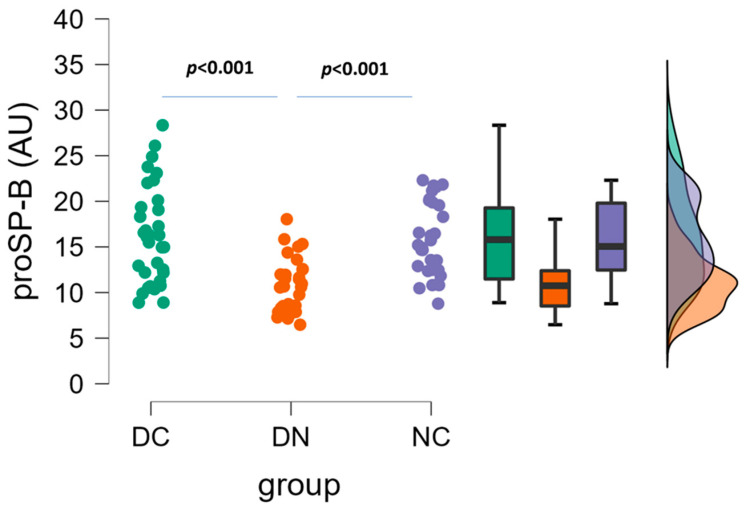
Plasma concentrations of proSP-B in the three groups of patients. Raincloud data plot, as well as box plots, and theoretical distributions are reported. Statistical differences were assessed by one-way ANOVA, followed by post hoc Tukey’s test.

ProSP-B plasma levels are illustrated in Figure 3. Significant differences (one-way ANOVA followed by Tukey’s post hoc test) were detected between DC and DN groups (16.1 ± 5.3 AU vs. 10.9 ± 2.9 AU, *p* < 0.001), as well as between DN and NC groups (10.9 ± 2.9 AU vs. 15.7 ± 4.0 AU, *p* < 0.001), because the mean values of the DN group were lower than those of the other two groups.

A correlation with age was evaluated for the three SPs. As Table S2 shows, no significant relationships were observed, both by the group of patients, and considering the patients altogether.

Regarding gender influence on the plasma level of SPs, since the number of female patients in the study groups is small, the comparison between SPs was performed by only considering male patients (Table S3). The overall profile of the three SPs’ proteins had results similar to those of the entire cohort, including the female subjects.

**Figure 4:**
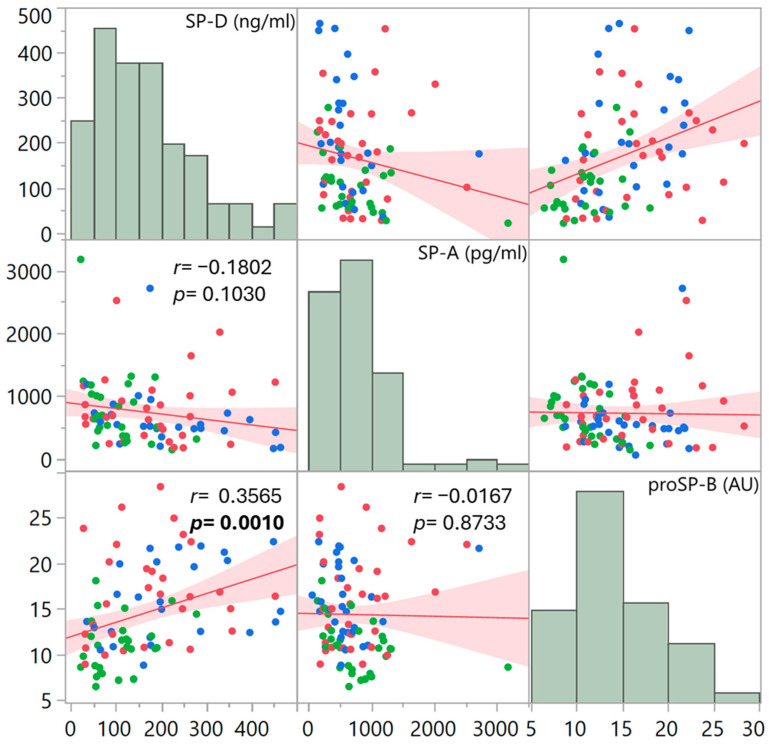
Scatter plot matrix showing pairwise correlations between the plasma surfactant protein levels, considering the three patient groups altogether (red points: DC; green points: DN; blue points: NC). Each dot plot presents the fitted linear regression line with confidence intervals of estimation. Pearson’s correlation coefficient *r* and its significance are indicated for each paired comparison. The upper and lower triangles of the matrix are mirrors of each other. The distribution of data is also presented as histograms.

In order to detect any correspondence between the surfactant proteins, the presence of a pairwise linear correlation was evaluated, considering the overall data of the three groups of patients (Figure 4). A significant correlation was only found between SP-D and proSP-B (*r* = 0.3565, *p* = 0.0010).

To establish the presence of links between surfactant proteins and glyco-oxidation parameters, including advanced glycation end-products (AGE), a receptor for advanced glycation end-products (RAGE), and its soluble form (sRAGE), we delved into existing data that highlighted diverse patterns within these patient groups [27]. As reported in Table S4, the only significant correlations occurred between sRAGE and proSP-B for DC patients (*r* = 0.3721, *p* = 0.0330), between sRAGE and SP-D for NC patients (*r* = 0.4040, *p* = 0.0452), and between RAGE and SP-D for DN patients (*r* = 0.4491, *p* = 0.0165).

**Figure 5:**
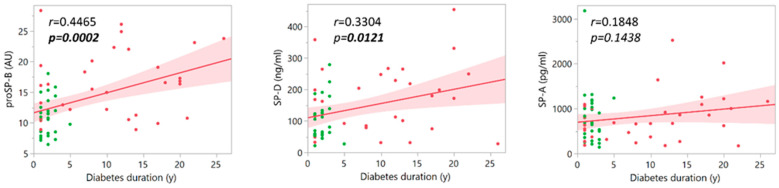
Pairwise correlations between the plasma surfactant protein levels and diabetes duration in the two groups of patients with diabetes (DC and DN), considered together (red points: DC; green points: DN).

A correlation was also tested between the duration of diabetes and the measured plasma surfactant proteins. As Figure 5 shows, considering DN and DC groups together, a significant correlation was detected for proSP-B (*r* = 0.4465, *p* = 0.0002) and SP-D (*r* = 0.3304, *p* = 0.0121). However, when data were analyzed for each patient group, the correlation of all surfactant protein levels with disease duration did not reveal any significant association (Table S5 and Figure S1).

**Figure 6:**
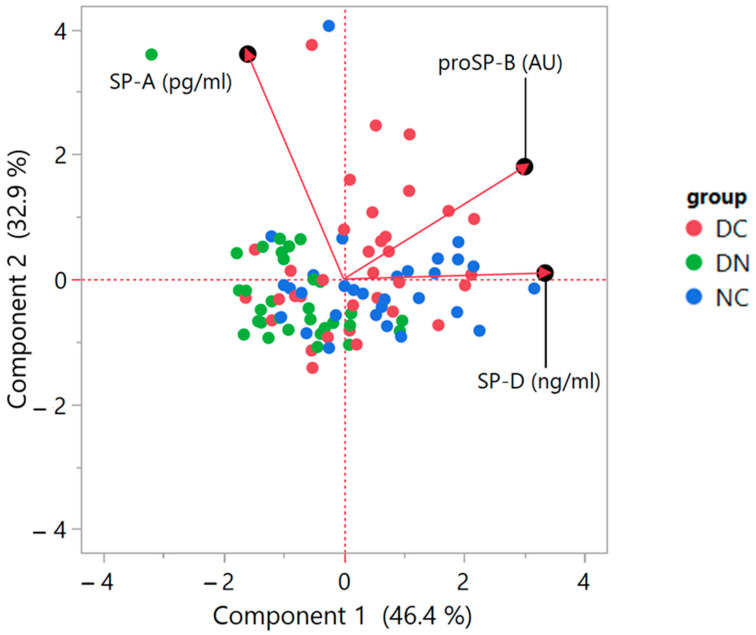
Biplot of the Principal Component Analysis (PCA) based on correlations. The graph indicates the PCA score plot with the location of single data points (red dots: DC; green dots: DN; blue dots: NC) together with the plot of loadings, showing the vectors representative of the variables of interest. The loading plot illustrates how the variables correlate with one another, given that a small angle implies positive correlation, a large one suggests negative correlation, and a 90° angle is indicative of absence of correlation between two variables. The axes legends show the Variance estimation with Residual/Restricted Maximum Likelihood (REML). For details on the interpretation of the data, please refer to [30,31].

A further statistical analysis was performed using the non-parametric Principal Component Analysis (PCA) method. As the biplot of Figure 6 shows, a correlation was detected between proSP-B and SP-D, as indicated by the small angle between the respective variable vectors of the loadings. Conversely, no correlation occurred between proSP-B and SP-A, suggested by the 90° angle between vectors; furthermore, a low correlation appeared between SP-A and SP-D levels, indicated by an angle that was not much further from 90°. The biplot showing the data scores also suggests a partial clustering of the three groups of patients, according to the behavior of the three analyzed surfactant proteins.

## 4. Discussion

The study uncovered that patients with T2D and CHD (DC group), along with those with CHD without diabetes (NC group), had higher levels of proSP-B and SP-D in their plasma compared to T2D patients (DN group). Additionally, the levels of proSP-B and SP-D were significantly correlated in all patients, as evidenced by both linear regression and non-parametric PCA analysis. Despite this, no differences in SP-A plasma levels were observed among the three subject groups. These findings provide information on a new possible biochemical signature for the risk of cardiovascular disease in individuals with T2D.

Notably, the plasma concentrations of the immature form of SP-B, namely proSP-B, were found to be more elevated in DC and NC patients, compared to DN patients with no history of CHD. Indeed, this result is in line with the findings by Gargiulo et al. [19], who detected increased levels of the immature forms of proSP-B in heart failure patients, indicating its potential use as a biological marker of alveolar-capillary membrane damage. A successive prospective clinical study by Magrì et al. [20] confirmed the findings, providing a strong relationship between circulating immature proSP-B levels, gas exchange alterations, and functional limitations in heart failure patients.

The plasma profile of proSP-B is similar to that of SP-D, also suggesting that this protein has an association with a clinical profile of cardiovascular pathology. SP-D is a protein that plays a vital role in protecting the lungs from infections. However, there is growing evidence that suggests it may also have implications for cardiovascular health [7,10,19,32]. While researchers are still exploring how SP-D influences heart health, they have discovered that it possesses both anti-inflammatory and pro-inflammatory properties [7,10]. Previous studies have primarily focused on the association between SP-D and pulmonary diseases, particularly chronic obstructive pulmonary disease (COPD). However, it is important to understand the role of SP-D in other pathologies, especially since recent research has shown a link to CHD [10], making it a potential therapeutic target. Indeed, research has found a significant correlation between circulating SP-D levels and the development of atherosclerosis and heart failure. Circulating SP-D could indicate the degree of atherosclerosis or the risk of cardiovascular-related mortality, potentially influencing the clinical outcome [7,10,33,34]. It has been reported that elevated SP-D levels are a good predictor of cardiovascular morbidity and mortality, adding prognostic information beyond traditional risk factors like age [7]. It is of note that both of our patient groups with CHD, whether they are affected by diabetes or not (DC and NC groups, respectively), have a similar age, therefore allowing a balanced comparison according to the impact of ageing. Moreover, patients did not present respiratory infections or a history of pulmonary disease or cancer, so this study did not consider all potential factors known to affect the expression of SPs. The current data suggest that there are increased levels of SP-D in the plasma of DC patients, similar to what was found in NC patients. This suggests that the protein may play a role as an indicator of CHD in T2D patients. While this study has found a significant association between SP-D and the clinical presence of a cardiovascular complication, it is not possible to determine any causal relationship due to the observational nature of the study. It could be that this is merely an epiphenomenon in the pathophysiological evolution of the chronic disease, or a consequence of altered translocation of the protein across the lung-capillary membrane [7].

SP-A is primarily known for its roles in the respiratory system, where it plays a crucial part in lung function. Surfactant proteins, including SP-A, have key roles in the respiratory system, particularly in maintaining lung surfactant, lung homeostasis, and lung function during acute lung injury [1,35]. While SP-D, a related protein, has been associated with cardiovascular disease in clinical studies, as reported above, there is no direct mention of SP-A’s involvement in cardiac conditions in the available literature. In particular, since there is limited direct information regarding the relationship between SP-A and cardiac function, the present study was aimed at determining the circulating level of this protein in a cardiovascular risk condition, such as T2D. The results appeared to exclude any relevant association between SP-A plasma concentration and the cardiovascular risk, since the values were not statistically different in all three groups of patients.

The soluble RAGE form (sRAGE) reflects the total concentration of RAGE present in cells and plasma [36]. Serum sRAGE levels are positively associated with the presence of CHD [37], regardless of the presence of diabetes [27]. In the present study, a positive correlation was detected between SP-D and proSP-B with s-RAGE in CHD patients, with and without diabetes (groups DC and NC, respectively), suggesting a possible link between serum levels of surfactant proteins and cardiovascular risk condition, because plasma levels of sRAGE may be associated with plaque vulnerability in patients with CHD, according to previous studies [38,39]. Our study shows that surfactant proteins are not affected by classical cardiovascular risk factors, particularly diabetes and dyslipidemia, but rather, it is cardiac pathology, both in terms of altered contractile function (HF) [19,20] and in the course of ischemic coronary artery disease, that induces a significant increase in serum levels, especially of the components most sensitive to vascular inflammation—in this context, the significant correlation of both SP-D and pro-SP-B with sRAGE levels—which are associated with plaque instability in CHD patients. Furthermore, the findings have a significant correlation between RAGE and SP-D for patients with diabetes, but no CHD (DN group), which may suggest a possible role as a parallel marker of vascular damage in the follow-up of vascular damage development.

Due to the observational nature of the sampling protocol, also related to our previous investigation [27], consisting of a single-point plasma measurement for each patient, it was not possible to estimate a time-dependent behavior of the plasma surfactant proteins. Therefore, we could not speculate on the timeline for SPs’ plasma concentrations, nor provide elucidation about a direct causative relationship with CHD. The correlations found here must therefore be taken as a possible association between the circulating SPs and the cardiovascular disease condition. The fact that patients were well-controlled with respect to diabetes and hypertension, and since no significant differences occurred in the use of antihypertensive drugs/aspirin/statins, allows us to exclude a specific role of these factors on SPs’ expression. The identification of the association of plasma SPs, specifically SP-D and proSP-B, with a cardiovascular risk profile of patients goes in line with our previous investigation on multiple circulating proteins [40], which have been suggested as possible biomarkers of cardiovascular damage progression associated with T2D, with promising classification results in terms of sensitivity and specificity.

As a further consideration, observing the two groups of patients affected by diabetes, with or without CHD, namely DC and DN groups, it is possible to speculate that the disease duration can significantly affect SP-D and proSP-B plasma levels. Here, the short history of diabetes in DN patients (affected by diabetes patients without CHD) represents an optimal comparison term for catching the proteomic pattern before the onset of cardiovascular complications, which occurs within several years, as documented by the DC group. Although the association with duration of disease is not evident by separately considering the two groups of patients with diabetes, possibly due to the limited number of patients, this observation may deserve attention in further studies.

The present investigation has limitations. The first is represented by the relatively small number of participants, due to the structure of the protocol related to our previous study [27], that did not rely on a specific power analysis as a preliminary evaluation. Another limitation is linked to the fact that the cohort comprises patients attending the local outpatient diabetes clinic, and therefore the sample is representative of a limited geographical area. Since this is a cross-sectional observational study, we could not evaluate every possible lifestyle factor or comorbidity, leaving the possibility of residual confounding factors. Additional limitations, strictly linked to the observational nature of the study, regard the difficulty to make a causal inference, or to fully interpret the associations with cardiovascular complications for the identified SPs. However, the significance obtained with the present data appears clear in order to show the role of the SPs in distinguishing patients with diabetes with or without CHD. The findings deserve confirmation with a larger sample size, and with a more specific experimental protocol, which will expand the evaluation of possible correlations between the identified SPs and other additional biomarkers, for instance, those related to inflammation and oxidative stress. The present investigation considered a long duration of diabetes and CHD, in order to have a reference point for which people with diabetes have an increased rate of CHD mortality, similar to that of subjects without diabetes but with a history of prior myocardial infarction, according to Wannamethee et al. [25]. The impact of different years of diabetes and CHD on SPs’ plasma levels remains to be investigated. Furthermore, the fact that patients with cancer and infections can have altered levels of SPs is a confounding condition that must be taken into account regarding the role of SPs as indicators of CHD in T2D. A further limitation regards the absence of a healthy control group, in order to have a comparison value of the SPs of interest in normal conditions. Gargiulo et al. [19], however, presented values of proSP-B in healthy subjects (11.65 ± 6.5 AU), which are comparable to those found in the DN group (*p* = 0.58), supporting the fact that an increase in the protein is typical of CHD’s features, where proSP-B plasma levels are higher.

In conclusion, the present study extends the knowledge on the role of plasma SPs’ levels, not only as already demonstrated predictors of lung diseases, but also as possible indicators of CHD in T2D. Although the present findings represent a single snapshot within the time course of diabetes vascular complications, the role of SPs’ profile is of interest, and remains to be fully elucidated in more detailed future investigations, specifically in people affected by diabetes.

## Data Availability

Data collected in the study will be made available using the data repository Zenodo (https://zenodo.org), upon request to the corresponding author.

## Supplementary Materials

The following supporting information can be downloaded at: https://www.mdpi.com/article/10.3390/life14070886/s1. Table S1: Clinical and metabolic parameters of subjects in the study; Table S2: Correlations between age and SPs in the three groups of patients, and altogether; Table S3: Plasma concentrations of SPs in the three groups of patients, considering only male subjects; Table S4: Correlation between the SP proteins and glyco-oxidation parameters in the three groups of patients; Table S5: Correlation between the plasma levels of SP proteins and diabetes duration in the two groups of patients with diabetes (DC and DN, respectively); Figure S1: Pairwise correlations between the plasma surfactant protein levels and diabetes duration.
