# Supplementary material for: Immature Surfactant Protein Type B and Surfactant Protein Type D Correlate with Coronary Heart Disease in Patients with Type 2 Diabetes
Source: Life (Basel). 2024 Jul 17;14(7):886. doi: 10.3390/life14070886 (PMC11277833; doi:10.3390/life14070886)
Supplement: Supplementary file 1 [file life-14-00886-s001.zip › Table S1 new.pdf]

Table S1. Clinical and metabolic parameters of subjects in study.

	**DC (n=34)**	**DN (n=31)**	**NC (n=30)**
Gender (M/F)	31/3	22/9	22/8
Age (y)	65.4±8.3**	60.0±5.9††	65.6±6.0
Diabetes duration (y)	9.9±7.6***	1.9±0.9	---
BMI (kg/m^2)	29.8±3.8	29.2±3.4	26.8±5.1‡
FPG (mg/dl)	141.6±41.1	144.8±32.1†††	94.7±12.9‡‡‡
HbA1c (%)	7.0±0.8	6.9±0.8†††	5.8±0.2‡‡‡
Total cholesterol (mg/dl)	152±34***	191±35†††	155±40
LDL cholesterol (mg/dl)	82±30**	112±39††	86±31
HDL cholesterol (mg/dl)	42±9	48±10	53±16‡‡
Triglycerides (mg/dl)	161±79	139±72†	91±40‡‡‡

Data are expressed as mean ±SD. For each continuous variable, symbols indicate significant differences between means by the Tukey's HSD test following one-way ANOVA. ***p<0.001, **p<0.01, DC vs DN; †††p<0.001, ††p<0.01, †p<0.05, DN vs NC; ‡ ‡ ‡ p<0.001, ‡ ‡ p<0.01, ‡ p<0.05, DC vs NC.

Abbreviations: BMI, body mass index; FPG, fasting plasma glucose; HbA1c, glycated haemoglobin; HDL, high density lipoprotein; LDL, low density lipoprotein.

Full information regarding patients' cohort has been previously published in Piarulli et al. (2022, 2024). Methods used for metabolic parameters determination are described in the mentioned papers.

[Piarulli F, Banfi C, Brioschi M, Altomare A, Ragazzi E, Cosma C, Sartore G, Lapolla A. The Burden of Impaired Serum Albumin Antioxidant Properties and Glyco-Oxidation in Coronary Heart Disease Patients with and without Type 2 Diabetes Mellitus. *Antioxidants* (Basel). 2022;11(8):1501. doi: 10.3390/antiox11081501.

Piarulli, F., Banfi, C., Ragazzi, E., Gianazza, E., Munno, M., Carollo, M., Traldi, P., Lapolla, A., & Sartore, G. Multiplexed MRM-based proteomics for identification of circulating proteins as biomarkers of cardiovascular damage progression associated with diabetes mellitus. *Cardiovascular Diabetology,* 2024; 23(1), 36. doi:10.1186/s12933-024-02125-1. CC BY 4.0]
