# Supplementary material for: Immature Surfactant Protein Type B and Surfactant Protein Type D Correlate with Coronary Heart Disease in Patients with Type 2 Diabetes
Source: Life (Basel). 2024 Jul 17;14(7):886. doi: 10.3390/life14070886 (PMC11277833; doi:10.3390/life14070886)
Supplement: Supplementary file 1 [file life-14-00886-s001.zip › Table S2 new.pdf]

Table S2. Correlations between age and SPs in the three groups of patients, and altogether.

Protein	By group			All patients
	DC	DN	NC	
SP-D (ng/ml)	*r*=-0.0207 (*p*=0.9153)	*r*=-0.1515 (*p*=0.4416)	*r*=0.0515 (*p*=0.8070)	*r*=0.1160 (*p*=0.2995)
SP-A (pg/ml)	*r*=0.1189 (*p*=0.5101)	*r*=0.0999 (*p*=0.5929)	*r*=0.0335 (*p*=0.8605)	*r*=0.0593 (*p*=0.5704)
proSP-B (AU)	*r*=-0.2134 (*p*=0.2331)	*r*=0.2094 (*p*=0.2668)	*r*=0.1916 (*p*=0.3103)	*r*=0.1422 (*p*=0.1738)
