# Supplementary material for: Immature Surfactant Protein Type B and Surfactant Protein Type D Correlate with Coronary Heart Disease in Patients with Type 2 Diabetes
Source: Life (Basel). 2024 Jul 17;14(7):886. doi: 10.3390/life14070886 (PMC11277833; doi:10.3390/life14070886)
Supplement: Supplementary file 1 [file life-14-00886-s001.zip › Table S3 new.pdf]

Table S3. Plasma concentrations of SPs in the three groups of patients, considering only male subjects.

Protein	group			One-way ANOVA	Tukey's test significant pairwise comparisons
	DC (*n*=31)	DN (*n*=22)	NC (*n*=22)		
SP-D (ng/ml)	164.7±101.9	116.6±69.4	198.1±112.2	p=0.039	DC-NC: p=0.031
SP-A (pg/ml)	796.5±538.3	756.2±650.2	600.5±536.4	p=0.666	---
proSP-B (AU)	15.5±4.9	11.1±3.0	16.2±4.1	p<0.001	DC-NC; p<0.001<br>DN-NC; p<0.001

Statistical differences were assessed by one-way ANOVA followed by post-hoc Tukey's test.
