# Supplementary material for: Immature Surfactant Protein Type B and Surfactant Protein Type D Correlate with Coronary Heart Disease in Patients with Type 2 Diabetes
Source: Life (Basel). 2024 Jul 17;14(7):886. doi: 10.3390/life14070886 (PMC11277833; doi:10.3390/life14070886)
Supplement: Supplementary file 1 [file life-14-00886-s001.zip › Table S4 new.pdf]

Table S4. Correlation between the SP proteins and glyco-oxidation parameters in the three groups of patients.

Group	Variable by variable		*r*	n	Lower 95%CI	Upper 95%CI	*P*
DC	AGE (µg/ml)	SP-D (ng/ml)	0.1366	29	-0.2420	0.4791	0.4797
		SP-A (pg/ml)	-0.2989	33	-0.5825	0.0494	0.0910
		proSP-B (AU)	0.0710	33	-0.2791	0.4044	0.6946
	S-RAGE (ng/ml)	SP-D (ng/ml)	0.2188	29	-0.1606	0.5419	0.2541
		SP-A (pg/ml)	0.1935	33	-0.1604	0.5034	0.2805
		proSP-B (AU)	0.3721	33	0.0330	0.6344	0.0330*
	RAGE (pg/ml)	SP-D (ng/ml)	0.2281	29	-0.1510	0.5487	0.2340
		SP-A (pg/ml)	0.0782	33	-0.2725	0.4105	0.6655
		proSP-B (AU)	0.2956	33	-0.0531	0.5800	0.0949
DN	AGE (µg/ml)	SP-D (ng/ml)	-0.2673	28	-0.5823	0.1175	0.1691
		SP-A (pg/ml)	0.0558	31	-0.3046	0.4022	0.7655
		proSP-B (AU)	-0.1665	30	-0.4969	0.2062	0.3793
	S-RAGE (ng/ml)	SP-D (ng/ml)	0.2358	28	-0.1505	0.5597	0.2270
		SP-A (pg/ml)	-0.0780	31	-0.4207	0.2842	0.6765
		proSP-B (AU)	-0.1536	30	-0.4869	0.2187	0.4176
	RAGE (pg/ml)	SP-D (ng/ml)	0.4491	28	0.0913	0.7042	0.0165*
		SP-A (pg/ml)	-0.1457	31	-0.4755	0.2200	0.4341
		proSP-B (AU)	0.1718	30	-0.2009	0.5011	0.3639
NC	AGE (µg/ml)	SP-D (ng/ml)	0.0720	25	-0.3326	0.4542	0.7323
		SP-A (pg/ml)	-0.0541	30	-0.4065	0.3122	0.7763
		proSP-B (AU)	-0.1276	30	-0.4664	0.2439	0.5018
	S-RAGE (ng/ml)	SP-D (ng/ml)	0.4040	25	0.0106	0.6891	0.0452*
		SP-A (pg/ml)	-0.0442	30	-0.3981	0.3212	0.8165
		proSP-B (AU)	0.3451	30	-0.0173	0.6274	0.0618
	RAGE (pg/ml)	SP-D (ng/ml)	0.3837	25	-0.0134	0.6763	0.0583
		SP-A (pg/ml)	-0.0857	30	-0.4326	0.2833	0.6524
		proSP-B (AU)	0.0499	30	-0.3160	0.4030	0.7932

*Asterisk indicates significant correlation.

AGE: advanced glycation end products; RAGE: receptor for advanced glycation end-products; sRAGE: soluble form of RAGE.

AGE, S-RAGE and RAGE data were presented in Piarulli et al. (2022). [Piarulli F, Banfi C, Brioschi M, Altomare A, Ragazzi E, Cosma C, Sartore G, Lapolla A. The Burden of Impaired Serum Albumin Antioxidant Properties and Glyco-Oxidation in Coronary Heart Disease Patients with and without Type 2 Diabetes Mellitus. Antioxidants (Basel). 2022 Jul 30;11(8):1501. doi: 10.3390/antiox11081501]
