# Supplementary material for: Immature Surfactant Protein Type B and Surfactant Protein Type D Correlate with Coronary Heart Disease in Patients with Type 2 Diabetes
Source: Life (Basel). 2024 Jul 17;14(7):886. doi: 10.3390/life14070886 (PMC11277833; doi:10.3390/life14070886)
Supplement: Supplementary file 1 [file life-14-00886-s001.zip › Table S5 new.pdf]

Table S5. Correlation between the plasma levels of SP proteins and diabetes duration in the two groups of patients with diabetes (DC and DN, respectively).

**Group**	**Variable by Variable**		***r***	***n***	**Lower 95%**	**Upper 95%**	***P***
**DC**	Diabetes duration (y)	SP-D (ng/ml)	0.1496	29	-0.2295	0.4893	0.4386
		SP-A (pg/ml)	0.3263	33	-0.0191	0.6022	0.0638
		proSP-B (AU)	0.2233	33	-0.1300	0.5263	0.2116
**DN**	Diabetes duration (y)	SP-D (ng/ml)	0.1474	28	-0.2388	0.4934	0.4541
		SP-A (pg/ml)	-0.1248	31	-0.4588	0.2402	0.5036
		proSP-B (AU)	0.0896	30	-0.2797	0.4358	0.6379
