# Supplementary figures and images for: Immature Surfactant Protein Type B and Surfactant Protein Type D Correlate with Coronary Heart Disease in Patients with Type 2 Diabetes
Source: Life (Basel). 2024 Jul 17;14(7):886. doi: 10.3390/life14070886 (PMC11277833; doi:10.3390/life14070886)

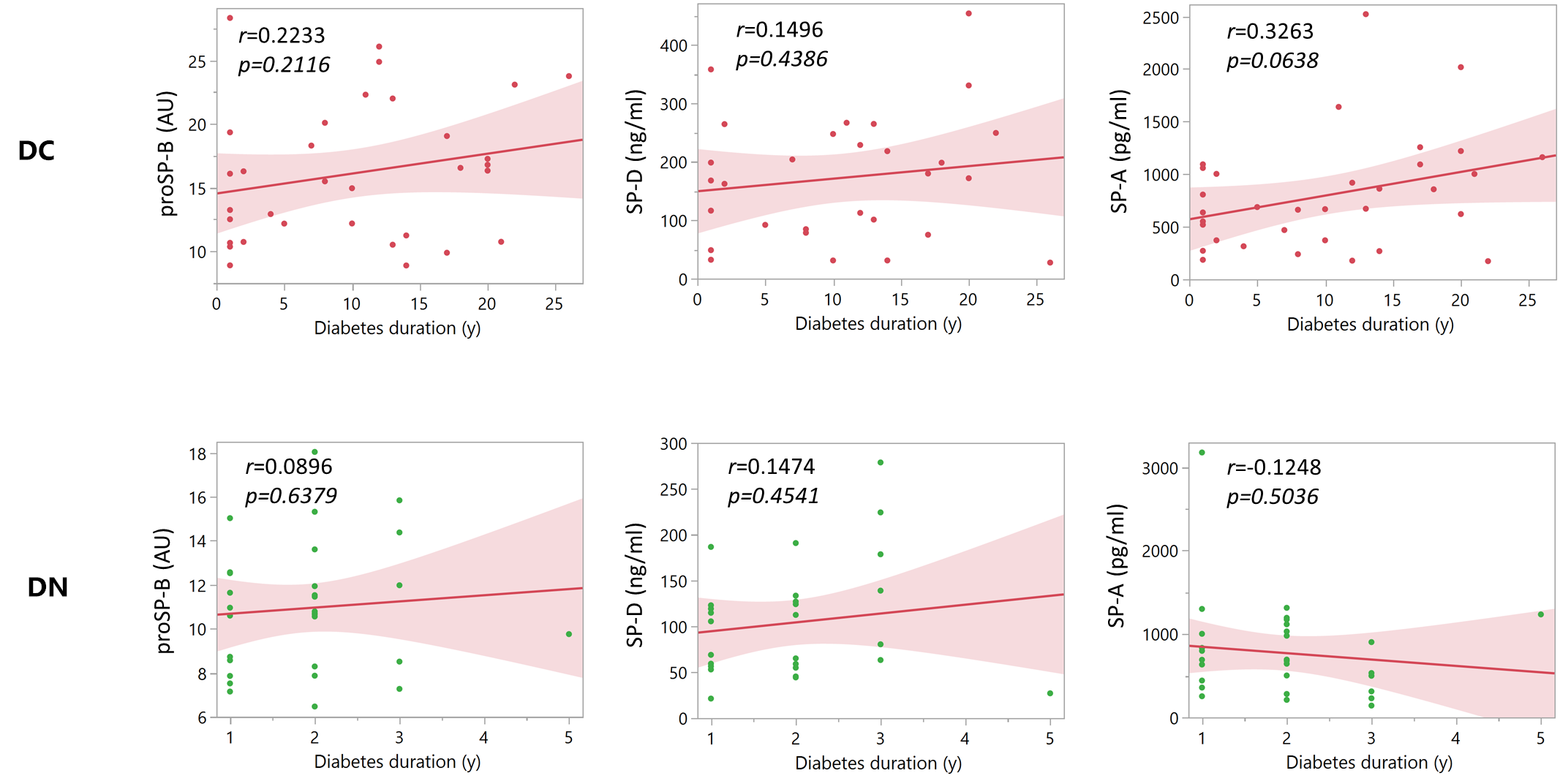

Supplement: Supplementary file 1 [file life-14-00886-s001.zip › Fig S1 new.tif]
